# *In vitro* Screening of Herbal Medicinal Products for Their Supportive Curing Potential in the Context of SARS-CoV-2

**DOI:** 10.1155/2022/8038195

**Published:** 2022-09-06

**Authors:** Hoai Thi Thu Tran, Philipp Peterburs, Jan Seibel, D. Abramov-Sommariva, Evelyn Lamy

**Affiliations:** ^1^Molecular Preventive Medicine, University of Freiburg, Medical Center and Faculty of Medicine, Engesserstraße 4, 79108 Freiburg, Germany; ^2^Bionorica SE, Kerschensteinerstraße 11-15, 92318 Neumarkt, Germany

## Abstract

COVID-19 herbal medicinal products may have the potential for symptom relief in nonsevere or moderate disease cases. In this *in vitro* study we screened the five herbal medicinal products Sinupret extract (SINx), Bronchipret thyme-ivy (BRO-TE), Bronchipret thyme-primula (BRO TP), Imupret (IMU), and Tonsipret (TOP) with regard to their potential to (i) interfere with the binding of the human angiotensin-converting enzyme 2 (ACE2) receptor with the SARS-CoV-2 spike S1 protein, (ii) modulate the release of the human defensin HBD1 and cathelicidin LL-37 from human A549 lung cells upon spike S1 protein stimulation, and (iii) modulate the release of IFN-*γ* from activated human peripheral blood mononuclear cells (PBMC). The effect of the extracts on the interaction of spike S1 protein and the human ACE2 receptor was measured by ELISA. The effects on the intracellular IFN-*γ* expression in stimulated human PBMC were measured by flow cytometry. Regulation of HBD1 and LL-37 expression and secretion was assessed in 25 d long-term cultured human lung A549 epithelial cells by RT-PCR and ELISA. IMU and BRO-TE concentration-dependently inhibited the interaction between spike S1 protein and the ACE2 receptor. SINx, TOP, and BRO-TE significantly upregulated the intracellular expression of anti-viral IFN-*γ* from stimulated PBMC. Cotreatment of A549 cells with IMU or BRO TP together with SARS-CoV-2 spike protein significantly upregulated mRNA expression (IMU) and release of HBD1 (IMU and BRO TP) and LL-37 (BRO TP). The *in vitro* screening results provide first evidence for an immune-activating potential of some of the tested herbal medicinal extracts in the context of SARS-CoV-2. Whether these could be supportive in symptom relief or curing from SARS-CoV-2 infection needs deeper understanding of the observations.

## 1. Introduction

All over the world, the population is struggling with the outbreak of COVID-19. Effective anti-viral therapies are unavailable so far, comprehensive vaccination has yet to be achieved, and various mutants of the enveloped, single-stranded RNA virus SARS-CoV-2 have emerged [1]. Thus, it is of critical importance to search for compounds being helpful to combat the viral pandemic. Testing the effectiveness of different drug types used previously in the treatment of other diseases is one strategy to accelerate the development. Herbal medicinal products have the potential to interfere with various steps of the viral replication cycle [2, 3]. Besides, they have been reported to exhibit an anti-inflammatory and immune-modulating potential and thus may also be supportive in preventing or attenuating mild to moderate SARS-CoV-2 infections [4–6]. In this pilot screening study, five herbal medicinal products, marketed for the treatment of respiratory infections [5–8], were explored for their potential to (i) interfere with the binding of the human angiotensin-converting enzyme 2 (ACE2) receptor with the SARS-CoV-2 spike protein. The ACE2 receptor, which has been found in various organs including type I and II pneumocytes, endothelial cells, oral and nasal mucosa, and also the intestinal tissues, liver, kidney, or brain [9, 10], has been identified as the key cellular receptor, facilitating the uptake of the SARS-CoV-2 virus into the host cell [11]. Drugs targeting the interaction between the spike protein receptor-binding domain of SARS-CoV-2 and the ACE2 receptor may thus offer some protection against this novel viral infection [12, 13]. (ii) We investigated whether the extracts can modulate the release of the defensin HBD1 and cathelicidin LL-37 from human A549 lung cells upon SARS-CoV-2 spike protein stimulation. In the innate immune system, these antimicrobial peptides (AMPs) have a nonenzymatic inhibitory effect on a broad spectrum of microorganisms [14]. Human beta defensins (HBDs) have known anti-viral effects on both enveloped and nonenveloped viruses [15]. Due to the low specificity of defensins compared the adaptive arm of the immune system, anti-viral applications of defensins are conceptually ideal for protection against different viral infections [14, 16], and they have also been discussed as potential valuable tools against SARS-CoV-2 infection [17, 18]. Moreover, LL-37 has been suggested as the SARS-CoV-2 attachment inhibitor. It was shown to bind to the SARS-CoV-2 RBD, resulting in competitive ACE-2 recruitment inhibition. In *in vitro* as well as in mice experiments, it could suppress SARS-CoV-2 spike pseudovirion infection [19]. (iii) We investigated whether the plant extracts could modulate the activated immune system in terms of regulating the anti-viral interferon gamma (IFN-*γ*) production. This type II interferon is produced by T lymphocytes and NK cells and is essential for anti-viral defence. It suppresses virus replication and activates T cell cytokine production [20]. High levels of IFN-*γ* have been detected in mild cases of COVID-19 with parasitic infections compared to the low levels in severe cases [21]. In another study, IFN-*γ* expression by CD4^+^ T cells tended to be lower in severe cases (14.1%) as compared to moderate cases (22.8%) [22].

## 2. Methods

### 2.1. Extracts

All plant extract mixtures were provided by Bionorica SE either in a dried or fluid form. The dried extracts were used in different dilutions. The following extracts (Bionorica SE, Neumarkt in der Oberpfalz, Germany) were investigated:

Bronchipret® thyme-ivy (BRO-TE) is an extract of the thyme herb (*Thymus vulgaris* L.) and ivy leaves (*Hedera helix* L.). BRO-TE is a mixture of fluid extracts of the thyme herb (extractant: ammonia solution 10% (*m/m*)/glycerol (85%) (*m/m*)/ethanol 90% (*v*/*v*)/water (1 : 20 : 70 : 109); drug-extract ratio (DER): 1 : 2–2.5) and ivy leaves (extractant: ethanol 70% (*v*/*v*); DER: 1 : 1) as contained in Bronchipret® syrup with a thyme/ivy fluid extract ratio of 10 : 1. In order to minimise the ethanol content in the test system, the extract mixture was dealcoholized by rotary evaporation to a final ethanol content of 1% (*v*/*v*). To control for the loss of volatile ingredients, specific identity tests were performed with the concentrate.

Bronchipret® thyme-primula (BRO TP), an extract of the thyme herb (*Thymus vulgaris* L.) and primula root (*Primula veris* L.), is a mixture of genuine dry extracts of the thyme herb (extraction solvent: ethanol 70% (*v*/*v*); DER: 6–10 : 1) and primula root (extractant: ethanol 47% (*v*/*v*); DER 6–7 : 1) as contained in Bronchipret® TP film-coated tablets without excipients, and with a final thyme/primula dry extract ratio of 2.67 : 1. A stock solution of 100 mg/ml was prepared in 50% EtOH.

Imupret® (IMU): 100 g Imupret oral drops contain 29 g of an ethanolic-aqueous extract (extraction solvent: ethanol 59 Vol.−%) out of Radix Althaeae 0.4 g, Flores Chamomillae 0.3 g, Herba Equiseti 0.5 g, Folia Juglandis 0.4 g, Herba Millefolii 0.4 g, Cortex Quercus 0.2 g, and Herba Taraxaci 0.4 g with a total-ethanol content of 19% (*v*/*v*). In order to minimize the ethanol content in the test system, the extract mixture was dealcoholized (>0.5% (*v*/*v*)) by rotary evaporation. The content quality of the dealcoholized test item complied with Imupret® oral drops was checked by identity tests and quantitative analysis.

Sinupret® extract (SINx): the dry extract combined the genuine dry extracts (BNO 1011) of gentian root, primula flower, sorrel herb, elder flower, and verbena herb with a ratio of 1: 3:3:3:3 (extraction solvent: ethanol 51% (*v*/*v*); DER 3–6 : 1) as contained in the Sinupret® extract coated tablets without excipients. A stock solution of 100 mg/ml was prepared in 50% EtOH.

Tonsipret® (TOP): homeopathic dilution in tablets contain 37.5% Dilution Capsicum D3, 37.5% Dilution Guajacum D3, and 25.0% mother tincture Phytolacca. In order to the minimise ethanol content in the test system, the mixture was dealcoholized (>0.5% (*v*/*v*)) by rotary evaporation. The quality of the dealcoholized test item complied with the corresponding manufacturing stage of the herbal medicinal product Tonsipret® was checked by identity analyses.

All the extracts were centrifuged (16.000 × *g*, 3 min, RT) and the supernatant was used for the experiments. The final concentration of the solvent (ethanol) was less than 0.5% in all assays.

### 2.2. Human A549 Lung Cell Line and Primary Human PBMC

The human lung adenocarcinoma A549 cell line was purchased from DSMZ (Braunschweig, Germany). For experiments, cells were used at a low passage number after thawing. Cells were cultured in a RPMI medium containing 10% heat-inactivated FCS, 1% penicillin/streptomycin, and 1% L-glutamine for 25 days for differentiation according to the protocol by Cooper et al., 2016 [23]. After 25 days, cells were exposed to the extracts for different time periods either with or without cotreatment with SARS-CoV-2 spike protein (trenzyme, Konstanz, Germany).

For PBMC isolation, blood was taken in Li-heparin vacutainers from healthy volunteers at the University of Freiburg-Medical Center after written informed consent. The study was approved by the Ethics Committee of the University of Freiburg and carried out according to their guidelines and regulations (ethical vote 373/20). PBMC isolation was done by centrifugation on a LymphoPrepTM gradient using SepMate centrifugation tubes (STEMCELL Technologies, Cologne, Germany). Cells were then washed twice with PBS, and viability and concentration were determined using the trypan blue exclusion test.

### 2.3. Assessment of SARS-CoV-2 Spike-ACE2 Binding Inhibition

The capacity of the extracts to inhibit the SARS-CoV-2-spike-ACE2 binding was tested using the SARS-CoV-2 (COVID-19) inhibitor screening kit (BioCat GmbH, Heidelberg, Germany) according to the manufacturer's instructions. This colorimetric ELISA assay measures the binding between immobilized the SARS-CoV-2 spike protein RBD and biotinylated human ACE2 protein. Colorimetric detection was carried out using streptavidin-HRP followed by TMB incubation.

### 2.4. Quantification of Human Defensins by qRT-PCR

A549 cells were treated with extracts with/without the SARS-CoV-2 spike S1 protein (trenzyme, Konstanz, Germany) for different time periods. Total RNA was isolated from the cells using the RNeasy mini-isolation kit from Qiagen (Hilden, Germany) with a purification step using the RNase-free Dnase kit (Qiagen) according to the manufacturer's instructions. The quantity and quality of RNA were determined by using a NanoDrop ND-1000 spectrophotometer (Thermo Fisher Scientific, Darmstadt, Germany). Change in mRNA expression levels was measured by qRT-PCR as described previously [24, 25]. Briefly, 1 *μ*g of total RNA was reversed-transcribed to cDNA using a RevertAid first strand cDNA synthesis kit (Thermo Fisher Scientific, Darmstadt, Germany). Next, the template cDNA, equal to 25 ng of total RNA, was used for each PCR reaction. Samples were analysed in 384-well plates using the Roche LightCycler 480 system at 95°C for 10 min, followed by 40 cycles (95°C for 20 s, 60°C for 30 s) and a final extension at 72°C for 45 s, followed by a standard melting curve analysis. The delta-delta Ct method was used to calculate the relative expression of mRNA [26]. Each qRT-PCR reaction was performed in triplicates, and each experiment was carried out at least three times, independently. GAPDH and b-tubulin were used as endogenous controls.

### 2.5. Quantification of HBD1 and LL-37 Peptide Release from A549 Cells

HBD1 and LL-37 peptide release was quantified in supernatants from 25-day long-term cultured A549 cells using a human BD-1 standard ABTS ELISA development kit (PeproTech, Hamburg, Germany) and LL-37 human ELISA kit (HycultBiotech, Beutelsbach, Germany) according to the manufacturers' instructions.

### 2.6. Flow Cytometric Analysis of Intracellular IFN-Gamma Expression

PBMC were treated with extracts for 6 h and costimulated with 50 ng/ml phorbol 12-myristate 13-acetate (PMA), 1 *μ*g/ml ionomycin, and a SARS-CoV-2 S peptide pool (Miltenyi Biotec, Germany) for an immune reaction together with 10 *μ*g/ml brefeldin A. Cells were then collected and processed for intracellular staining with an anti-IFN-*γ*-FITC monoclonal antibody (Miltenyi Biotec, Bergisch Gladbach, Germany). Changes in the subset of lymphocyte cells and cytokine production (IFN-*γ*) were assessed using flow cytometry (FACSCalibur, BD, Heidelberg, Germany).

### 2.7. Determination of Cell Viability

The LIVE/DEAD fixable far red dead cell stain kit (Thermo Fisher Scientific, Germany) and the LDH-Glo cytotoxicity assay (Promega, Gutenbergring, Germany) were used to determine cytotoxicity of extracts in PBMC and A549 cells, respectively, after 24-hour exposure, according to the manufacturers' instructions.

### 2.8. Statistics

Results were analysed using the GraphPad Prism 6.0 software (La Jolla, California, USA). Data were presented as means + SD. Statistical significance was determined by the one-way ANOVA test followed by Holm–Sidak's correction. *p* values < 0.05 (^*∗*^) were considered statistically significant and <0.01 (^*∗∗*^) were considered highly statistically significant.

## 3. Results

### 3.1. Effect of Extracts on SARS-CoV-2-Spike-ACE2 Binding Inhibition

**Figure 1:**
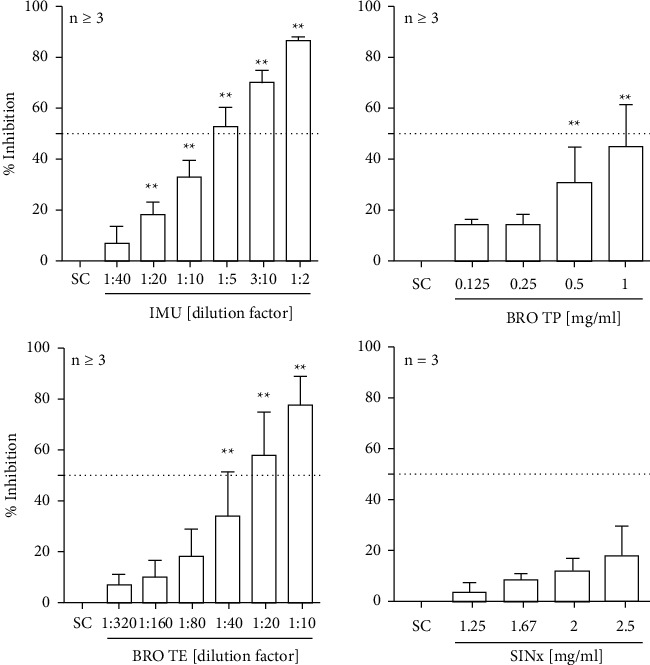
Efficacy of spike-ACE2 binding inhibition by the tested extracts. The SARS-CoV-2 S protein RBD coated plate was incubated with plant extracts and biotinylated human ACE2 for 1 h. Thereafter, the plate was washed thoroughly and incubated with streptavidin-HRP followed by colorimetric detection using a multiplate reader from Tecan (Germany). Bars are means + SD of at least three independent experiments. ^*∗*^*p* < 0.05 and ^*∗∗*^*p* < 0.01. Significance of difference was calculated relative to the respective solvent control by one-way ANOVA.

The potential of the extracts to inhibit the interaction of the SARS-CoV-2 spike protein and the ACE2 receptor was tested in a cell free assay. A concentration-dependent inhibitory effect on spike binding to ACE2 was seen with extract IMU (maximum of 86% ± 1.3), extract BRO TP (maximum of 44% ± 16.8), and extract BRO TE (maximum of 77% ± 10.8). For extract TOP, no relevant effect (data not shown), and for SINx, minor effects were observed (Figure 1).

### 3.2. Effect of Extracts on Intracellular IFN-*γ* Expression in Activated Human PBMC

**Figure 2:**
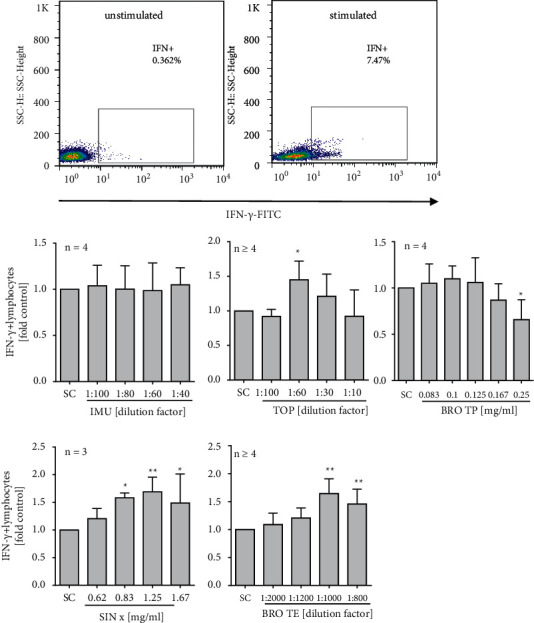
Intracellular IFN-*γ* expression from stimulated human PBMC upon extract treatment. Cells were stimulated with a mixture of SARS-CoV-2 S peptide pool, PMA, and ionomycin, and treated with the extracts for 6 hours. Brefeldin A was used to enhance intracellular cytokine staining signals. Representative scattergrams of intracellular IFN-*γ* staining without (a) and after (b) stimulation are given. Bars are means + SD of at least three independent experiments. ^*∗*^*p* < 0.05 and ^*∗∗*^*p* < 0.01. Significance of difference was calculated relative to the respective solvent control by one-way ANOVA.

First, cytotoxicity of the extracts on human PBMCs was quantified after 24 h exposure. Relevant toxicity (i.e. >10%) was evident for extract IMU at ≥1 : 40 dilution, for extract BRO TP at ≥0.25 mg/ml, and for BRO-TE at ≥1 : 200 dilution. For TOP and SINx, no relevant toxicity could be seen in the applied *in vitro* model. Upon stimulation with a mixture of SARS-CoV-2 S peptide pool, PMA, and ionomycin, a significant increase in the intracellular IFN-*γ* expression (in the range of 7–15%) could be observed (Figure 2). Compared to control cells, a significant increase in IFN-*γ* expression could be seen in stimulated PBMC with extract TOP (145% ± 27 at a 1 : 60 dilution), extract SINx (169% ± 26 at 1.25 mg/ml), and extract BRO-TE (157% ± 31 at a 1 : 1000 dilution) (Figure 2). For extract IMU and BRO TP, no additional increase in the IFN-*γ* expression was seen. In contrast, at ≥0.167 mg/ml, extract BRO TP had an inhibitory effect on IFN-*γ* expression, which is likely due to beginning cytotoxicity.

### 3.3. Effects of Extracts on mRNA Expression and Peptide Release of HBD1 and LL-37

**Figure 3:**
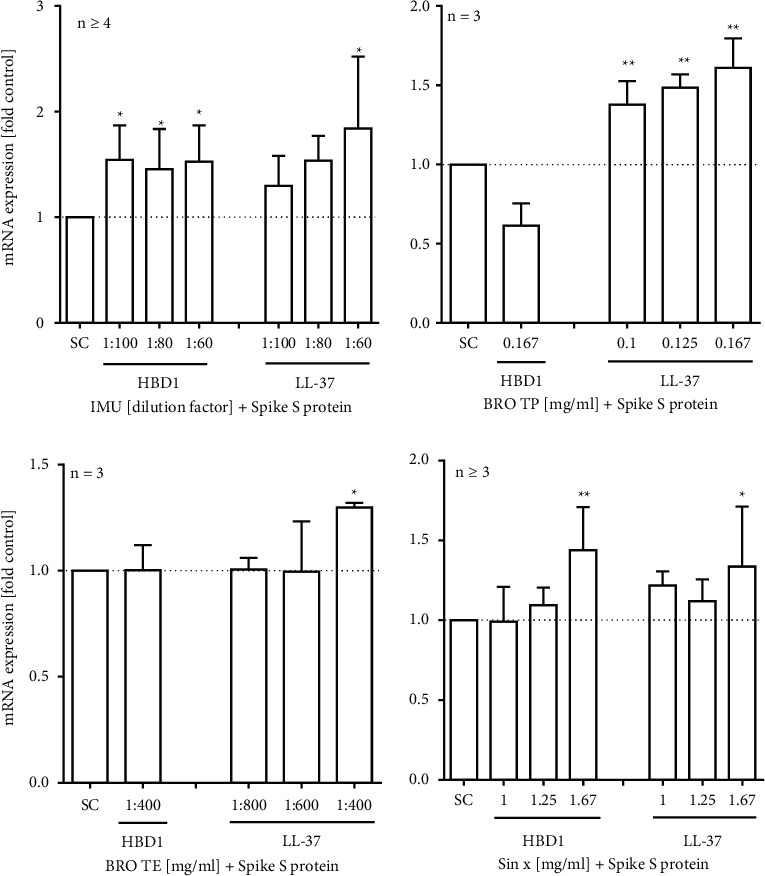
Effect of extracts on HBD1 and LL-37 mRNA expression in spike protein-stimulated A549 lung cells. A549 cells were cultured for 25 days and cotreated with extracts and SARS-CoV-2 spike S1 protein for 24 h. Bars are mean values + SD of at least three independent experiments. ^*∗*^*p* < 0.05 and ^*∗∗*^*p* < 0.01. Significance of difference was calculated relative to the respective solvent control by one-way ANOVA.

**Figure 4:**
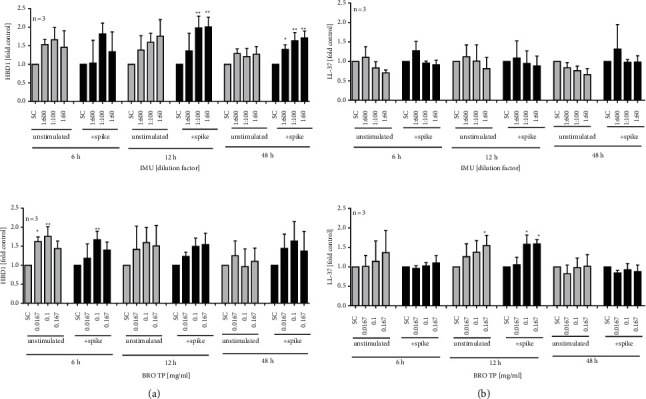
Effects of extracts on HBD1 and LL-37 peptide secretion measured by the ELISA technique. Human A549 lung cells were cultured for 25 days and exposed to (a) IMU or (b) BRO TP for different time periods with and without SARS-CoV-2 spike S1 protein stimulation. Bars are mean values + SD of at least three independent experiments. ^*∗*^*p* < 0.05 and ^*∗∗*^*p* < 0.01. Significance of difference was calculated relative to the respective solvent control by one-way ANOVA.

For first insights into the effects of the test items on cellular defence mechanisms, we analysed the mRNA expression and secretion of HBD-1 and LL-37 in human A549 cells. Based on the findings in human PBMC, the absence of relevant cytotoxicity (i.e., >10%) upon extract exposure was first confirmed in 25 d long-term cultured A549 cells using the LDH-Glo cytotoxicity assay (data not shown). We then treated the cells with SARS-CoV-2 spike S1 protein together with the extracts for 24 h. Thereafter, a significant increase in the HBD1 mRNA expression was measured for IMU (154% ± 33 at 1 : 100 dilution) and extract SINx (144% ± 27 at 1.67 mg/ml) as compared to control (Figure 3). A similar trend was observed in LL-37 regulation upon cotreatment with IMU (184% ± 68 at 1 : 60 dilution), BRO TP (161% ± 17 at 0.167 mg/ml), or BRO-TE (130% ± 2 at 1 : 400 dilution) together with the SARS-CoV-2 spike protein. Additionally, the extract-induced secretion of HBD1 and LL37 was analysed by ELISA in the presence and absence of the spike protein at different time points (Figure 4). Both extracts, IMU and BRO TP, could also significantly trigger the secretion of HBD1 peptide from A549 cells with or without S1 spike protein cotreatment. Extract BRO TP could also significantly trigger the secretion of LL-37 either with S1 spike protein co-treatment or without. This was not the case for extract IMU.

## 4. Discussion

Herbal extracts are known to induce diverse cellular defence mechanisms following viral infection of human cells [27, 28]. Using different screening assays, we show here that extracts of marketed herbal medicinal products elicit potential beneficial effects *in vitro* in terms of cellular defence activation upon a challenge with the SARS-CoV-2 spike protein or peptide mix.

The first step of viral infection is the interaction of the virus with the host cell. In case of SARS-CoV-2 the spike protein interacts with the ACE2 receptor on the surface of epithelial cells, e.g., in the oral cavity or respiratory tract [3, 10, 29]. In our study, extracts from IMU and BRO-TE were able to interfere with the binding between the S1 spike protein and the human ACE2 receptor. Computational binding studies showed that common herbal secondary metabolites such as luteolin or quercetin could be able to bind and block the ACE2 receptor, and also bind the SARS-CoV-2 spike protein [3, 27, 30]. A demonstration of the interference with SARS-CoV-2 spike–ACE2 binding using cell-free or *in vitro* assays is missing for most of them. So, for quercetin and its metabolites, inhibition of recombinant human ACE2 (rhACE2) activity has been reported *in vitro* [31]. The rhACE2 activity was inhibited by rutin, quercetin-3-O-glucoside, tamarixetin, and 3, 4-dihydroxyphenylacetic acid by 42–48%. With an IC50 of 4.48 *μ*M, quercetin was the most potent rhACE2 inhibitor tested in this study. The herbal extracts investigated here contain high amounts of these plant metabolites [5, 6, 32–35]. Thus, these constituents could account for or add to the inhibitory effects observed for the two herbal extract mixtures IMU and BRO-TE and it will be important to further ascertain this hypothesis, which is currently being investigated in ongoing studies. The products are mixtures made from several medicinal plants. Therefore, it might also be helpful to determine the plant with the largest share on this effect. This becomes even more relevant, since the inhibition was observed at a concentration range which turned out to be cytotoxic to human PBMC, thus questioning the biological relevance of the observations for the complex plant extract mixtures. However, another study reported some anti-viral activity of Bronchipret TP against SARS-CoV-2, by reducing the SARS-CoV-2 RNA load in a noncytotoxic concentration range, which indicates a certain potential of this extract to restrict SARS-CoV-2 replication in cells [36].

Anyway, IMU and BRO TP also activated the human innate immune defence by increasing the level of defensin HBD1 and/or cathelicidin LL-37 upon SARS-CoV-2 spike protein stimulation, and this was evident at much lower concentrations. In addition, LL-37, consisting of 37 amino acids and an overall positive net charge (+6), can eliminate microbes directly by electrostatic binding to negatively charged molecules on microbial membranes [37]. HBD-1 is another integral part of innate immune protection, shielding mucosal surfaces from microbial challenges [38]. By regulating chemokine and cytokine production, both AMPs help to maintain homeostasis of the immune system and display anti-viral properties, as for an example evidenced by gene regulation upon viral challenge or expression in cells involved in viral defence [14]. In secretions from the lung and nose, it was found that LL-37 could reach high concentrations, indicating a relevant role for LL-37 in lung immune defence mechanisms [39–41]. Defensins can be detected in the mucosa of all respiratory tissues, including the pharynx [22, 23]. The effect of AMPs on virus infections appears to be specific for the virus, AMP, as well as the target cell. First evidence for LL-37 has been reported, showing its binding to the SARS-CoV-2 spike protein and its inhibitory action on the binding of the spike protein to its entry receptor using binding competition studies [37, 42]. In another study, a high structural similarity between LL-37 and the N-terminal helix of the receptor-binding domain of SARS-CoV-2 was reported [43]. Using *in vitro* and *in vivo* experiments, LL-37 was recently confirmed to suppress SARS-CoV-2 spike pseudovirion infection by binding to SARS-CoV-2 RBD, resulting in competitive ACE-2 recruitment inhibition [19]. LL-37 has even been proposed for the treatment of COVID-19 patients by some researchers [44]. Thus, stimulating LL-37 expression by the herbal extracts might have several advantages during the early phase of SARS-CoV-2 entry, but this hypothesis, again, needs further verification.

In the present study BRO-TE and SINx extracts could be shown to further boost the immune response of PBMC in terms of intracellular IFN-*γ* expression, which was activated by a mixture including SARS-CoV-2 spike peptides. Once a virus has entered the cell and is replicated, a host immune response starts to combat viral infection [29]. An early interferon response of the host has been reported to be essential for an effective defence against SARS-CoV-2 infection [29, 45]. In turn, a decrease of IFN-*γ* positive T-helper cells might increase the risk for severe courses of COVID-19 [46, 47]. The here reported *in vitro* findings provide a first hint that BRO TE and SINx may help to further increase IFN-*γ* expression during infection. On the one hand, IFN-*γ* is essential for anti-viral defence, but on the other hand persistent high levels of INF-*γ* have been reported to worsen the systemic inflammation, intensifying tissue injury and organ failure [48] during the COVID-19 disease. This ambiguous role of IFN-*γ* in the course of SARS-CoV-2 infection asks for special attention also in the here presented data.

In conclusion, the marketed herbal medicinal products tested in this study demonstrated a range of potential mechanisms to support the human immune defence against SARS-CoV-2 infection. Whether the observed effects could be relevant for the systemic use in man or limited to local effects, e.g., in the oral cavity, needs to be investigated. Further confirmatory mechanistic studies are necessary to gain a deeper understanding of the reported observations and the transferability of the *in vitro* results to the clinical situation needs to be tested.

## Data Availability

The datasets used and/or analysed during the current study are available from the corresponding author upon request.

## Abbreviations

ACE2: Angiotensin-converting enzyme 2
AMP: Antimicrobial peptide
BRO-TE: Bronchipret® thyme-ivy
BRO TP: Bronchipret® thyme-primula
CC_50_: Cytotoxic concentration
COVID-19: Coronavirus disease 2019
DER: Drug-extract ratio
IMU: Imupret®
PMA: Phorbol-12-myristat-13-acetat
PBS: Phosphate-buffered saline
FCS: Fetal calf serum
qRT-PCR: Quantitative reverse transcriptase polymerase chain reaction
SARS-CoV-2: Severe acute respiratory syndrome coronavirus 2
SD: Standard deviation
SINx: Sinupret extract®
TOP: Tonsipret®.
